# Prognostic Value of 48-Hour Biomarker Reassessment Beyond Admission SOFA for 28-Day Mortality in Sepsis

**DOI:** 10.3390/diagnostics16101522

**Published:** 2026-05-18

**Authors:** Norberth-Istvan Varga, Adela Benea, Vasile Hachi, Flavia Ignuta, Madalina-Ianca Suba, Mirela Turaiche, Maria Daniela Mot, Florin George Horhat

**Affiliations:** 1 Doctoral School, Department of General Medicine, “Victor Babeş” University of Medicine and Pharmacy, Eftimie Murgu Square, No. 2, 300041 Timișoara, Romania; norberth.varga@umft.ro (N.-I.V.); adela.benea@umft.ro (A.B.); 2 Department of Nursing, “Victor Babeş” University of Medicine and Pharmacy, Eftimie Murgu Square, No. 2, 300041 Timișoara, Romania; 3 Multidisciplinary Research Center on Antimicrobial Resistance (MULTI-REZ), “Victor Babeş” University of Medicine and Pharmacy, Eftimie Murgu Square, No. 2, 300041 Timișoara, Romania; horhat.florin@umft.ro; 4 “Victor Babes” Infectious Diseases and Pneumoftiziology Hospital, 300310 Timisoara, Romania; vasile.hachi@yahoo.com (V.H.); flavia.ignuta@umft.ro (F.I.); madalina.suba@umft.ro (M.-I.S.); 5 Department XIII, Discipline of Infectious Diseases, “Victor Babeş” University of Medicine and Pharmacy, Eftimie Murgu Square, No. 2, 300041 Timișoara, Romania; 6 Department of General Medicine, “Vasile Goldis” Western University of Arad, B-dul Revolutiei, No. 96, 310025 Arad, Romania; mot.dana@uvvg.ro; 7 Microbiology Department, “Victor Babeş” University of Medicine and Pharmacy, Eftimie Murgu Square, No. 2, 300041 Timișoara, Romania

**Keywords:** sepsis, sepsis biomarkers, C-reactive protein, procalcitonin, lactate, SOFA score, mortality

## Abstract

**Background/Objectives**: Sepsis is clinically dynamic, and isolated admission biomarker values may insufficiently capture early biological evolution after treatment initiation. This study evaluated whether routine biomarker reassessment at approximately 48 h provides incremental prognostic information beyond admission Sequential Organ Failure Assessment (SOFA) score for 28-day mortality in sepsis. The analysis was framed as an exploratory 48 h landmark prognostic assessment among patients who were alive and had complete biomarker reassessment data at 48 ± 6 h. **Methods**: We conducted a prospective single-center observational cohort study including adult patients with sepsis. Clinical and laboratory data were collected at baseline (M1) and repeated 48 ± 6 h later (M2). The primary outcome was 28-day mortality. Candidate biomarkers included C-reactive protein (CRP), procalcitonin (PCT), lactate (LAC), and neutrophil-to-lymphocyte ratio (NLR). PCT clearance and NLR change were calculated as relative changes between M1 and M2, whereas 48 h CRP and 48 h lactate were evaluated as early reassessment values. Exploratory logistic regression models were constructed using admission SOFA as the clinical reference model. Model discrimination and fit were summarized using receiver operating characteristic analysis, likelihood-ratio testing, and Nagelkerke R^2^; the models were not intended as validated individual-level risk calculators. **Results**: The 48 h landmark analytical cohort included 126 patients, of whom 44 (34.9%) died within 28 days. Admission biomarker values showed limited prognostic signal. SOFA alone showed fair discrimination (AUC 0.740). Among the primary SOFA-augmented models, SOFA plus PCT clearance showed the highest discrimination and explanatory performance (AUC 0.810; Nagelkerke R^2^ 0.332) and significantly improved model fit compared with SOFA alone. SOFA plus NLR change and SOFA plus 48 h lactate also provided incremental prognostic information, although their gains were more modest. In exploratory combined modeling, SOFA plus PCT clearance and NLR change provided the most coherent additional signal, with all predictors retaining independent associations with 28-day mortality. **Conclusions**: In this exploratory single-center 48 h landmark analysis, selected routine biomarker reassessment measures were associated with 28-day mortality beyond admission SOFA. PCT clearance provided the clearest incremental prognostic signal, while NLR change offered complementary information. Persistent 48 h lactate elevation was also informative, whereas lactate clearance was not. These findings should be interpreted as hypothesis-generating and require validation in larger cohorts, ideally including serial organ dysfunction measures such as 48 h SOFA or SOFA change.

## 1. Introduction

Sepsis is a life-threatening organ dysfunction caused by a dysregulated host response to infection and remains one of the most urgent and challenging conditions encountered in acute care medicine [1,2]. Despite advances in antimicrobial therapy, organ support, and protocolized resuscitation, sepsis continues to account for a substantial proportion of global morbidity and mortality, with an estimated burden of tens of millions of cases and millions of deaths worldwide each year [3,4]. Its clinical course is often rapidly progressive: an initially localized infection may evolve within hours into systemic inflammation, endothelial dysfunction, microcirculatory failure, and multiorgan injury, ultimately leading to septic shock and death if appropriate treatment is delayed or inadequate [1,3,5]. From a pathophysiological perspective, sepsis reflects not only an exaggerated inflammatory response, but also profound disturbances in coagulation, metabolism, immune regulation, and tissue perfusion, making early recognition and early risk stratification central to patient outcomes [2,5].

Because sepsis is biologically heterogeneous and clinically dynamic, prognostic assessment in routine practice has relied on a combination of severity scores and laboratory biomarkers. Clinical tools such as the Sequential Organ Failure Assessment (SOFA) score provide an established framework for quantifying acute organ dysfunction and remain central to bedside risk stratification [1,2]. At the same time, routinely available biomarkers, including C-reactive protein (CRP), procalcitonin (PCT), lactate (LAC), and the neutrophil-to-lymphocyte ratio (NLR), capture complementary aspects of the septic response, ranging from acute-phase inflammation and infection-related signaling to tissue hypoperfusion and immune dysregulation [5,6,7,8,9]. Increasingly, however, the literature suggests that isolated baseline values may be less informative than the trajectory of these markers over time. In particular, studies of PCT, CRP and lactate kinetics have shown that early decreases in biomarker levels are often more closely associated with survival than admission measurements alone, supporting the view that dynamic biomarker assessment may better reflect treatment response and evolving biological recovery [10,11,12,13,14].

Nevertheless, important gaps remain in the current literature. A large number of candidate biomarkers have been investigated in sepsis, including interleukin-6, presepsin, soluble triggering receptor expressed on myeloid cells-1, soluble urokinase-type plasminogen activator receptor, monocyte distribution width, and other host-response markers, but many of these remain primarily research-oriented, are not universally available, or are less practical for rapid and repeated use in routine care [6,15,16,17,18]. By contrast, biomarkers such as CRP, PCT, LAC, and NLR are inexpensive, rapidly obtainable, and already incorporated into routine clinical workflows in most hospitals worldwide, making them more attractive candidates for clinically applicable prognostic models. However, even among these accessible biomarkers, the literature remains fragmented: many studies have focused on single markers, have emphasized admission values rather than early trajectories, or have evaluated biomarker dynamics without embedding them in parsimonious models anchored to clinical severity. A clinically useful prognostic reassessment strategy should be simple, rapid, affordable, and based on variables that are already available in routine care; however, such strategies require careful validation before they can be used for individual-level risk prediction or clinical decision-making. Therefore, we ought to study small, biologically coherent combinations of accessible biomarkers and/or clinical severity scores that reflect distinct domains of sepsis pathophysiology, such as infection resolution, tissue perfusion recovery, and immune normalization.

In this context, the present study aimed to evaluate whether routinely available biomarkers, particularly selected early reassessment measures obtained at approximately 48 h, provide incremental prognostic information beyond admission SOFA score for 28-day mortality in sepsis. Because the biomarker variables of interest required repeat measurement, the analysis was conceptualized as an exploratory 48 h landmark prognostic assessment among patients who were alive and had complete biomarker data at both M1 and M2. Specifically, we compared the prognostic contribution of admission biomarker values, 48 h biomarker values, and selected relative biomarker changes, and explored whether small biologically coherent combinations of routine biomarkers could add information beyond baseline clinical severity. The study was not designed to develop or validate a clinical risk calculator, but rather to generate hypotheses for future validation studies.

## 2. Materials and Methods

### 2.1. Study Design, Setting, and Ethics

This study was designed as a prospective, observational, single-center cohort study conducted at the “Victor Babeș” Clinical Hospital of Infectious Diseases and Pulmonology, Timișoara, Romania. Patients were enrolled from the infectious disease inpatient setting; ICU transfer and organ-support interventions were recorded when they occurred during hospitalization, but the cohort was not designed as an ICU-only sepsis cohort. Consecutive adult patients admitted with sepsis during the study period were screened for eligibility. Data were collected over a 12-month period, from 1 June 2024 to 1 June 2025. The primary objective was to evaluate whether selected routine biomarker reassessment measures obtained at approximately 48 h provide incremental prognostic information beyond admission SOFA for 28-day mortality in sepsis. Because these biomarkers required repeat measurement, the analysis was framed as an exploratory 48 h landmark prognostic assessment rather than as an admission-time prediction model or a validated clinical risk calculator.

This study was conducted in accordance with the principles of the Declaration of Helsinki and was approved by the local institutional ethics committee of the participating hospital (approval number: 4808, from 4 June 2024). Written informed consent was obtained from all participants or, when appropriate, from a family member or legally authorized representative. All collected data were handled confidentially and entered into an anonymized research database accessible only to the study team.

### 2.2. Study Population

Eligible participants were adults admitted with suspected or documented infection and sepsis as defined by Sepsis-3 [1], i.e., infection-associated acute organ dysfunction represented by an increase in total SOFA score of ≥2 points. The diagnosis of infection was established by the treating clinical team based on microbiological, imaging, clinical, or source-specific evidence, as applicable.

Patients were included if they had (1) a diagnosis of sepsis, (2) available outcome data regarding 28-day mortality, (3) signed informed consent, and (4) sufficient clinical and laboratory information for the predefined analyses. Patients were excluded if 28-day outcome status was unavailable, if the admission diagnosis did not fulfill the prespecified study definition of sepsis, or if required data for the predefined complete-case analyses were unavailable. In the final analytical cohort, patients with incomplete data were excluded and a complete-case approach was used.

Because the main biomarker variables required both M1 and M2 measurements, the analytical cohort should be interpreted as patients with sepsis who had complete biomarker reassessment data at 48 ± 6 h. Patients who died before the M2 sampling window or lacked required M2 biomarker data could not contribute to the dynamic biomarker analysis.

### 2.3. Data Collection

Clinical and laboratory data were collected prospectively during hospitalization using standardized case documentation and hospital medical records. The study database included demographic data (age and sex), comorbidity burden, source of infection, admission clinical status, and serial laboratory measurements. Clinical variables included the Charlson Comorbidity Index (CCI), Glasgow Coma Scale (GCS), and SOFA score at admission. Infection foci were recorded according to the treating team’s clinical diagnosis and could include more than one site when applicable. Admission SOFA was calculated from the earliest available clinical and laboratory data at presentation by investigators using standard SOFA criteria; when chronic organ dysfunction was documented before the index infection, this was considered in baseline SOFA assessment. Laboratory measurements included white blood cell count (WBC), neutrophil-to-lymphocyte ratio (NLR), hemoglobin, platelet count, serum creatinine, C-reactive protein (CRP), procalcitonin (PCT), and lactate (LAC). Blood samples were processed in the hospital’s central laboratory using routine standardized automated methods.

M1 was defined as the first biomarker measurement obtained at hospital admission or at clinical sepsis recognition, corresponding to the baseline sample used for study enrollment. M2 was defined as the repeat biomarker measurement obtained 48 ± 6 h after M1 sampling. The M2 time point, therefore, represents a reassessment landmark rather than an admission-time predictor.

Lactate values were standardized to mmol/L before analysis; values originally recorded in mg/dL were converted to mmol/L. NLR was calculated as the ratio of absolute neutrophil count to absolute lymphocyte count from the same complete blood count.

After enrollment, patients or family members who agreed to participate provided contact information for outcome ascertainment. Vital status at 28 days after admission was determined through hospital records and, when necessary, by structured telephone follow-up with the patient or family member. All collected information was entered into a dedicated anonymized database and checked for internal consistency before analysis.

Derived variables were subsequently calculated from these two measurements as described below.

### 2.4. Outcomes, Biomarkers, and Derived Variables

The primary outcome of the study was 28-day mortality, coded as a binary variable (1 = death, 0 = survival). The principal candidate biomarkers evaluated in relation to this outcome were CRP, PCT, and LAC, selected because of their wide routine availability and their relevance to inflammation, infection response, and tissue hypoperfusion in sepsis. In addition, NLR was examined as a biologically plausible indicator of immune-response dynamics.

For each biomarker, admission values (M1) and approximately 48 h values (M2) were considered. The 48 h time point was selected a priori before data analysis as a clinically pragmatic interval for early reassessment. This interval was considered long enough to allow measurable changes in inflammatory, infectious, perfusion-related, and immune-cell biomarkers after initiation of therapy, while still being early enough to preserve the intended role of the models as early prognostic tools. Dynamic variables were defined a priori as early relative changes between these two time points. CRP clearance, PCT clearance, and lactate clearance were calculated using the formula “Clearance = ((M1 − M2)/M1) × 100”, so that higher values indicated a greater decline from the baseline. NLR change was calculated using the same approach: “NLR change = ((NLR M1 − NLR M2)/NLR M1) × 100”, with higher values indicating greater early decline or normalization of NLR.

In the multivariable analyses, admission SOFA was used as the baseline clinical severity anchor. Candidate biomarkers were selected based on routine clinical availability and biological relevance to sepsis pathophysiology. For each biomarker domain, the component carried forward into the primary exploratory models was chosen using clinical plausibility together with the observed descriptive and univariate signal. This strategy was exploratory and clinically guided, rather than a prespecified prediction-model development procedure or automated variable-selection process. The primary SOFA-augmented models, therefore, evaluated 48 h CRP, PCT clearance, 48 h lactate, and NLR change. Additional exploratory combined models assessed whether pairing PCT clearance with either NLR change or 48 h lactate provided further information beyond SOFA plus PCT clearance. These models were used to evaluate incremental prognostic associations and were not intended to generate a deployable individual-level prediction tool.

### 2.5. Statistical Analysis and Software

Continuous variables were assessed for distributional normality using the Shapiro–Wilk test. This study was designed as an exploratory prognostic analysis of 48 h biomarker reassessment rather than as the development or validation of a clinical risk calculator. The multivariable models were used to assess incremental prognostic information beyond admission SOFA, not to generate a deployable individual-level prediction tool. No formal sample-size calculation for prediction-model development was performed; the final sample size was determined by the number of eligible patients prospectively enrolled during the predefined study period.

Because the analyzed continuous variables showed non-normal distributions, they were summarized as median [25th–75th interquartile range], whereas categorical variables were reported as absolute frequencies and percentages. Between-group comparisons (survivors vs. non-survivors at 28 days) were performed using the Mann–Whitney U test for continuous variables and the Pearson chi-square test for categorical variables.

To assess crude associations with 28-day mortality, univariate logistic regression models were fitted for each predictor of interest, and results were reported as odds ratios (ORs) with 95% confidence intervals (95% CIs) and *p*-values. CRP was modeled per 10 mg/L increase, whereas clearance variables and NLR change were modeled per 10% increase to facilitate clinical interpretation. For the main prognostic analyses, multivariable logistic regression models were constructed using SOFA as the clinical base model. Candidate biomarker components were then added individually in parsimonious models to evaluate their incremental prognostic value beyond SOFA. Exploratory combined biomarker models were subsequently fitted by combining SOFA, PCT clearance, and one additional dynamic biomarker.

Model performance was evaluated by receiver operating characteristic (ROC) curve analysis and the corresponding area under the curve (AUC) with 95% confidence intervals. Overall model explanatory performance was summarized using Nagelkerke R^2^. Nested models were compared using the likelihood ratio test. All analyses were performed on complete-case data, and no imputation procedures were applied. A two-sided *p*-value < 0.05 was considered statistically significant.

Model discrimination and fit statistics were interpreted descriptively and comparatively within this exploratory cohort. Although AUC confidence intervals were estimated using nonparametric bootstrap resampling, bootstrap optimism correction, calibration assessment, shrinkage estimation, and decision-curve analysis were not performed. Therefore, the reported models should not be interpreted as validated prediction tools.

All statistical analyses were performed using SPSS version 26, IBM Corp., Armonk, NY, USA.

## 3. Results

### 3.1. Descriptive Analysis of the Study Cohort

**Figure A1:**
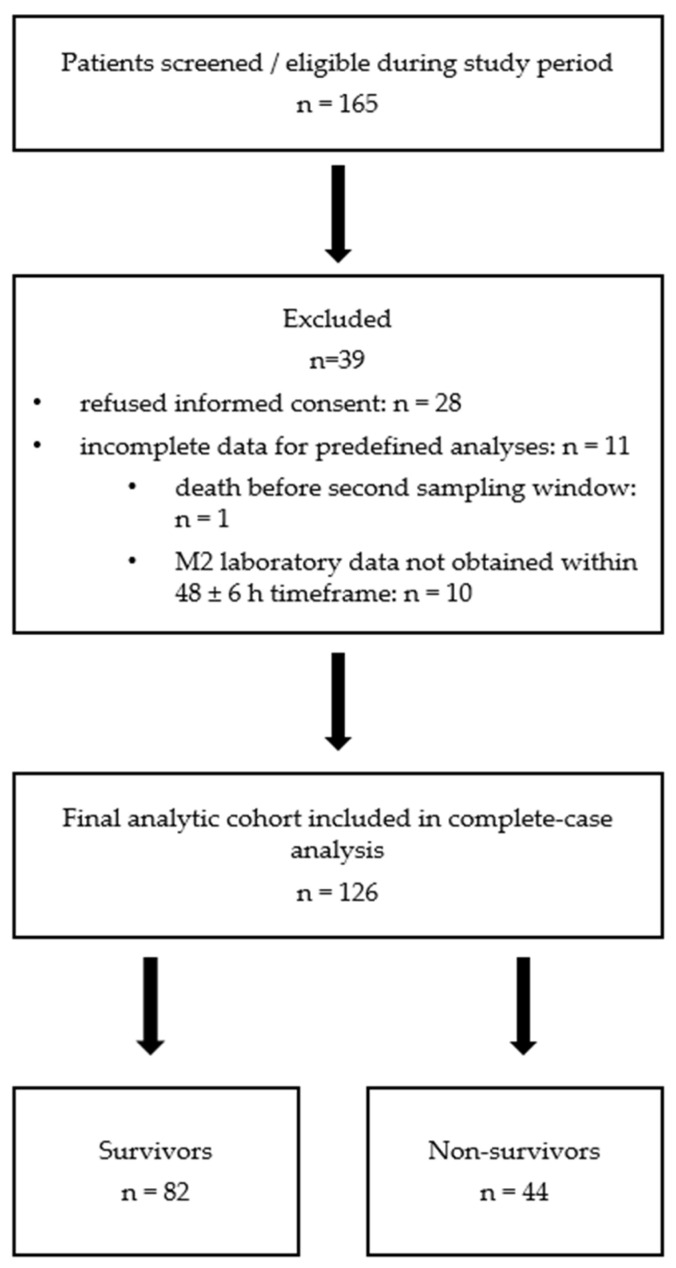
Study Flow Diagram.

From a total of 165 eligible patients, 28 refused to sign informed consent for participation, and 11 patients were excluded from the complete-case biomarker reassessment analysis because of missing required data, predominantly missing M2 biomarker data or incomplete 28-day follow-up. These 11 excluded patients included one patient who died before the predefined 48 h second sampling window and therefore could not contribute to the dynamic biomarker analysis, as shown in Appendix A Figure A1.

The final complete-case reassessment cohort included 126 patients, of whom 82 (65.1%) were classified as survivors (S) and 44 (34.9%) as non-survivors (NS). The median age of the overall cohort was 75 [64–80] years. The cohort included 70 women (55.6%) and 56 men (44.4%). Male sex was less frequent among non-survivors than among survivors, although this difference was not statistically significant (34.1% vs. 50.0%; *p* = 0.127).

The most frequent sites of infection were the urinary tract (52 cases, 41.3%), abdomen (43 cases, 34.1%), lungs (37 cases, 29.4%), and skin and soft tissue (25 cases, 19.8%). Because some patients had multiple infectious foci, infection-source categories were not mutually exclusive.

Overall, the cohort was characterized by a substantial burden of comorbidity, as reflected not only by the Charlson Comorbidity Index but also by the high prevalence of chronic cardiovascular and metabolic disease. The most common comorbidities were arterial hypertension (89 cases, 70.6%), diabetes mellitus (43 cases, 34.1%), dementia and heart failure (30 cases each, 23.8%), followed by atrial fibrillation and previous stroke (29 cases each, 23.0%). Immunocompromised status was documented in 12 patients (9.5%), mainly due to active malignancy (9 cases, 7.1%). Acute kidney injury was documented, according to the treating team’s clinical diagnosis and available hospital documentation, in 17 cases (13.5%) during the early hospitalization period, including 12 (14.6%) in the survivor group and 5 (11.4%) non-survivors. Renal replacement therapy was required in 4 patients (3.2%).

At presentation, non-survivors were significantly older than survivors (*p* = 0.037). Non-survivors had significantly higher SOFA scores at admission than survivors (*p* < 0.001). In contrast, although CCI values were numerically higher among non-survivors, this difference did not reach statistical significance (*p* = 0.075). Glasgow Coma Scale scores at admission were broadly similar between the groups and did not differ significantly (*p* = 0.102).

At admission (M1), non-survivors had significantly higher NLR values than survivors (9.28 [8.11–10.60] vs. 7.11 [6.15–8.92]; *p* < 0.001). By contrast, admission WBC, CRP, PCT, and LAC levels were not significantly different between the groups. Hemoglobin, platelet count, and serum creatinine also did not differ significantly at baseline. At 48 h after admission (M2), NLR remained significantly higher in non-survivors (8.83 [7.07–10.34] vs. 6.28 [5.56–7.69]; *p* < 0.001). LAC was also higher among non-survivors at M2 (4.19 [3.02–6.39] vs. 3.18 [2.12–4.80] mmol/L; *p* = 0.013), whereas WBC, CRP, and PCT remained comparable between the groups.

Although the cohort was managed primarily in an infectious disease inpatient setting, patients who required escalation of care were also followed after transfer to the intensive care unit. Overall, 15 patients required ICU transfer (11.9%). Organ-support requirements reflected a clinically heterogeneous sepsis population: 13 patients received vasopressor support (10.3%), 9 required invasive mechanical ventilation (7.1%), 14 received high-flow oxygen therapy (11.1%), and 4 underwent renal replacement therapy (3.2%).

Table 1 presents an overview of the cohort.

### 3.2. Univariate Associations with 28-Day Mortality

In univariate logistic regression analysis, higher admission SOFA score, higher M1 and M2 NLR, higher M2 CRP, and higher corrected M2 LAC were associated with increased odds of 28-day mortality. Specifically, admission SOFA score was associated with higher mortality risk (OR 1.56 per point, 95% CI 1.25–1.93, *p* < 0.001), as were admission NLR (OR 1.86 per unit, 95% CI 1.45–2.39, *p* < 0.001), NLR at 48 h (OR 2.05 per unit, 95% CI 1.56–2.69, *p* < 0.001), 48 h CRP (OR 1.05 per 10 mg/L, 95% CI 1.01–1.10, *p* = 0.028), and corrected 48 h lactate (OR 1.25 per 1 mmol/L, 95% CI 1.05–1.49, *p* = 0.014). Greater PCT clearance (OR 0.89 per 10% increase, 95% CI 0.83–0.96, *p* = 0.001) and greater NLR change (OR 0.66 per 10% increase, 95% CI 0.45–0.97, *p* = 0.033) were inversely associated with 28-day mortality. By contrast, age, sex, CCI, admission CRP, admission and 48 h PCT, admission lactate, and CRP and lactate clearance were not significantly associated with 28-day mortality in the univariate analysis. These results are summarized in Table 2.

### 3.3. Primary Multivariable Prognostic Models Beyond SOFA

The primary exploratory models assessed whether selected 48 h biomarker reassessment variables provided incremental prognostic information beyond admission SOFA in the 48 h landmark analytical cohort. Admission biomarker values were not prioritized for these models because the central aim was to evaluate early reassessment rather than admission-time biomarker prediction. The selected reassessment components were 48 h CRP, PCT clearance, 48 h lactate, and NLR change. Because model construction was clinically guided but partly informed by observed biomarker signals, the resulting comparisons should be interpreted as exploratory rather than confirmatory.

The results of the primary multivariable models are summarized in Table 3. SOFA remained independently associated with 28-day mortality across all models.

Adding CRP at 48 h to SOFA (Model 1) produced only a minimal increase in discrimination compared with SOFA alone and did not significantly improve model fit (AUC 0.751 vs. 0.740; likelihood ratio *p* = 0.152), while CRP M2 was not independently associated with mortality.

Model 2, combining SOFA with PCT clearance, showed the best overall performance, with the highest discrimination (AUC 0.810, 95% CI 0.721–0.885) and the highest Nagelkerke R^2^ (0.332), and significantly improved fit over SOFA alone (*p* < 0.001); both SOFA and PCT clearance remained significant in this model.

Model 3, which incorporated LAC M2, significantly improved model fit beyond SOFA, although the gain in discrimination was modest (AUC 0.770, 95% CI 0.687–0.854; likelihood ratio χ^2^ = 4.278, *p* = 0.039). In this model, both SOFA and LAC M2 remained independently associated with 28-day mortality.

Model 4, combining SOFA with NLR change, significantly improved fit compared with SOFA alone (AUC 0.765, 95% CI 0.664–0.855; likelihood ratio *p* = 0.021), and both SOFA and NLR change remained independently associated with 28-day mortality.

Overall, Model 2 provided the best discrimination and explanatory performance among the primary biomarker-augmented models. LAC M2 and NLR change also provided significant incremental information beyond SOFA, although their gains in discrimination were more modest. SOFA alone remained the most parsimonious clinical model.

**Figure 1:**
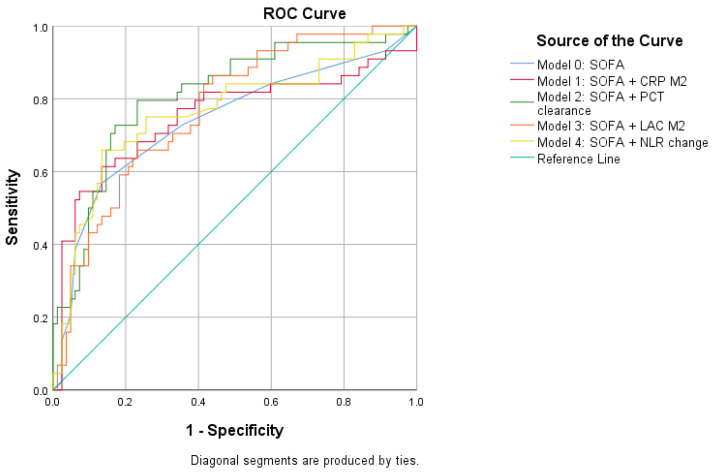
ROC Curves of the primary multivariable prognostic models for 28-day mortality. Model 0 included admission SOFA alone. Model 1 included SOFA and 48 h CRP. Model 2 included SOFA and PCT clearance. Model 3 included SOFA and 48 h LAC. Model 4 included SOFA and NLR change.

The ROC curves for the primary multivariable models are shown in Figure 1.

### 3.4. Exploratory Combined Biomarker Models Beyond SOFA

**Figure 2:**
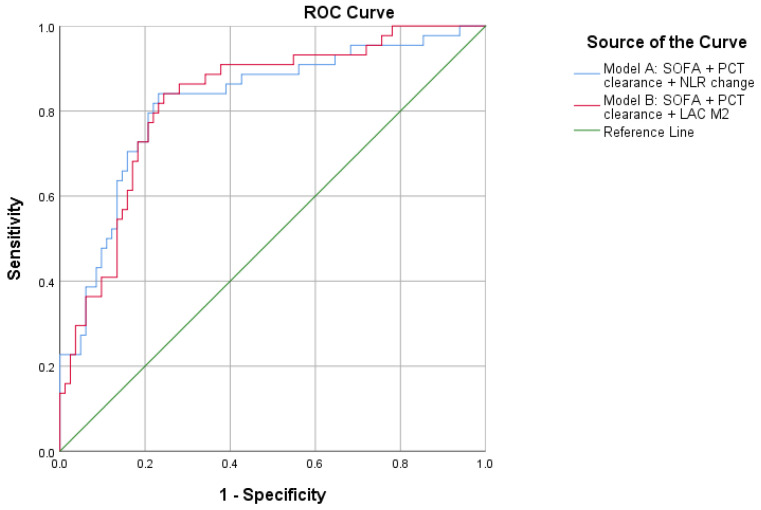
ROC curves of the exploratory combined biomarker models for 28-day mortality. Exploratory Model A included SOFA, PCT clearance, and NLR change. Exploratory Model B included SOFA, PCT clearance, and M2 LAC.

To further explore whether the prognostic value of PCT clearance could be enhanced by combining it with a complementary dynamic biomarker, two additional multivariable models were constructed beyond SOFA: one including NLR change (Exploratory Model A) and one including M2 LAC values (Exploratory Model B). The results of these exploratory models are summarized in Table 4, and their ROC curves are shown in Figure 2.

Both exploratory models showed higher discrimination than SOFA alone. Exploratory Model A, combining SOFA, PCT clearance, and NLR change, achieved an AUC of 0.821 (95% CI 0.733–0.895) and a Nagelkerke R^2^ of 0.378. In this model, all three predictors remained independently associated with 28-day mortality, and the model provided significant incremental value beyond the previous best primary model, SOFA plus PCT clearance (likelihood ratio *p* = 0.017).

Exploratory Model B, combining SOFA, PCT clearance, and LAC M2, achieved a numerically similar AUC of 0.826 (95% CI 0.751–0.902) and a Nagelkerke R^2^ of 0.353. In this model, SOFA and PCT clearance remained independently associated with mortality, whereas LAC M2 did not retain independent significance. The model did not significantly improve fit beyond SOFA plus PCT clearance (likelihood ratio χ^2^ = 2.548, *p* = 0.110).

Taken together, these findings suggest that the additional value of a second reassessment biomarker depended on whether it contributed information distinct from SOFA and PCT clearance. Although the model including 48 h lactate had a numerically similar AUC, lactate did not retain independent significance after PCT clearance was included. In contrast, NLR change remained independently associated with mortality and significantly improved model fit beyond SOFA plus PCT clearance. Therefore, within this exploratory cohort, the combination of admission SOFA, PCT clearance, and NLR change appeared to provide the most coherent prognostic reassessment signal. This finding requires external validation and should not be interpreted as a ready-to-use clinical prediction model.

## 4. Discussion

### 4.1. Interpretation of Findings

This study sought to determine whether selected routine biomarker reassessment measures provide incremental prognostic information beyond admission SOFA score for 28-day mortality in sepsis, and to identify which biomarker components are most informative when incorporated into parsimonious multivariable models. Because the main biomarker variables required repeat measurement, the findings should be interpreted as applying to patients with complete 48 h biomarker reassessment data rather than as admission-time prediction for all patients presenting with sepsis. More specifically, the analysis aimed to distinguish between biomarker measurements that merely reflect baseline inflammatory burden and those that more meaningfully capture early biological response during the first 48 h of hospitalization. Model selection was not performed through an indiscriminate search across multiple arbitrary combinations, but was instead guided by a stepwise and biologically reasoned strategy. Candidate biomarker components were first screened through the descriptive and univariate analyses, and only those showing either a measurable prognostic signal or strong clinical plausibility were carried forward into multivariable modeling. This is important from both a methodological and a clinical standpoint. Methodologically, it reduced unnecessary model proliferation, although overfitting and optimism remain possible given the modest number of events [19,20]. Clinically, it ensured that each selected model reflected a meaningful dimension of sepsis pathophysiology rather than a purely data-driven association.

In this framework, admission biomarker values were not prioritized in the primary models because they showed limited prognostic contribution and because the study focused on early reassessment beyond admission SOFA. The primary biomarker-augmented models, therefore, evaluated 48 h CRP, PCT clearance, 48 h lactate, and NLR change. SOFA alone already provided fair discrimination, and the addition of PCT clearance yielded the clearest improvement among the individual biomarker-augmented models, with the highest AUC and Nagelkerke R^2^. Further, 48 h LAC and NLR change also provided statistically significant incremental information beyond SOFA, although their gains in discrimination were more modest. However, the absolute increase in AUC should be interpreted as moderate rather than transformative. The main value of PCT clearance in this cohort was not that it replaced clinical severity assessment, but that it provided statistically significant and biologically plausible incremental information beyond admission SOFA. The model-building strategy was intentionally parsimonious and clinically guided, but it remained exploratory and partly informed by observed biomarker signals. Therefore, the relative performance of the models should be interpreted as hypothesis-generating rather than confirmatory.

From a biological perspective, the observed pattern is clinically coherent and helps explain why some models performed better than others. PCT clearance may be interpreted as a dynamic marker of infection-response evolution and early biological response, although it should not be considered a direct causal measure of treatment effectiveness in this observational study. NLR change reflects a different but complementary process, namely, early normalization of the host immune response; 48 h lactate reflects persistent tissue hypoperfusion or metabolic stress after early treatment, whereas lactate clearance did not provide a significant independent signal in this cohort. This rationale motivated the exploratory combined models, which were intentionally restricted to small, conceptually focused combinations rather than expanded into a broad search across many biomarker permutations. Notably, the combination of SOFA, PCT clearance, and NLR change produced the most convincing exploratory signal: all included predictors remained independently associated with mortality, and the model significantly improved fit beyond the previous best primary model. Although the model combining SOFA, PCT clearance, and M2 LAC showed a numerically similar AUC, M2 LAC did not retain independent significance and the model did not significantly improve fit beyond SOFA plus PCT clearance. Taken together, these findings suggest that, in this cohort, combining admission SOFA with PCT clearance and NLR change may offer a coherent exploratory reassessment signal, whereas 48 h lactate appeared informative mainly when added individually to SOFA.

Although no formal prospective sample size calculation was used to determine recruitment, the models were intentionally kept parsimonious relative to the number of observed events. With 44 deaths in the final cohort, the primary models remained parsimonious, corresponding to approximately 22 events per predictor in the two-predictor models and 14.7 events per predictor in the three-predictor exploratory models, thereby reducing the risk of overfitting. Nevertheless, given the modest cohort size and the exploratory nature of the analysis, these findings should be interpreted as hypothesis-generating.

### 4.2. Comparison with Previous Literature

Our findings support the growing view that, in sepsis, early biomarker trajectories may be more informative than isolated admission values for prognostic assessment. This pattern is consistent with the broader biomarker literature, which increasingly suggests that static baseline measurements often provide limited incremental information once clinical severity has been taken into account, whereas dynamic changes may better reflect the evolving interaction between infection control, host response, and early treatment adequacy [6,12,15,21]. In our cohort, admission CRP, PCT, and LAC values were not retained in the primary multivariable models, whereas 48 h CRP, PCT clearance, 48 h LAC, and NLR change emerged as the most plausible early reassessment candidates. This general hierarchy is in line with prior work showing that baseline biomarker levels may correlate with illness burden yet remain insufficiently discriminative for outcome prediction when used in isolation [6,12,13,15,22,23]. By contrast, repeated measurements and early biomarker kinetics appear to capture clinically meaningful biological evolution during the first days of sepsis management, when prognosis may still be modifiable.

Among the routine biomarkers evaluated, PCT clearance provided the clearest incremental prognostic signal beyond SOFA, which is broadly consistent with previous studies of procalcitonin kinetics. In the MOSES study, failure of PCT to decline substantially over the first days of illness was associated with increased 28-day mortality, while other cohorts similarly reported that early PCT kinetics predicted short-term outcome more effectively than baseline concentrations alone [11,12,22,23]. Our results extend this line of evidence by showing that, within a parsimonious SOFA-based model, PCT clearance not only improved discrimination but also remained independently associated with 28-day mortality. This supports the interpretation of PCT clearance as a marker that may reflect infection-response evolution and early biological response. However, antimicrobial appropriateness and source-control success were not systematically adjudicated, so PCT clearance should not be interpreted as a direct measure of treatment adequacy. In contrast, CRP at 48 h showed only limited incremental value in our primary models. Also, 48 h LAC retained independent prognostic significance when added to SOFA, supporting the relevance of persistent lactate elevation after early treatment. However, when corrected 48 h LAC was added to the exploratory model already containing SOFA and PCT clearance, it did not retain independent significance and did not significantly improve model fit. This does not necessarily contradict the literature, since previous studies have shown prognostic value for both lactate persistence and lactate kinetics in selected septic populations [24,25]. Rather, it suggests that in our cohort, the lactate signal was clinically relevant but partly overlapped with clinical severity and other early biological-response markers.

The exploratory models further suggest that combining biologically complementary reassessment variables may improve prognostic assessment more effectively than combining markers that overlap conceptually. NLR has been increasingly recognized as a simple and accessible index of immune dysregulation, integrating neutrophil-driven inflammation with relative lymphocyte suppression [7,26,27,28,29,30]. In our study, the addition of NLR change to SOFA and PCT clearance provided the most convincing exploratory signal, with all included predictors remaining independently associated with mortality and the combined model significantly improving fit beyond the previous best primary model. This finding is also consistent with prior reports showing that NLR, particularly when combined with SOFA, can enhance prognostic discrimination in sepsis [7,26]. By contrast, adding 48 h LAC to SOFA and PCT clearance yielded a numerically similar AUC but did not significantly improve fit and did not preserve independent significance for the lactate component. Taken together, these observations suggest that a model combining clinical severity with a marker of infection-response evolution and a marker of immune normalization may better represent early sepsis trajectory than combinations that do not add sufficiently distinct prognostic information.

From a practical perspective, this is particularly relevant because all biomarkers evaluated in our study are inexpensive, rapidly available, and already embedded in routine care, in contrast to many emerging sepsis biomarkers that remain promising but less accessible for real-world bedside application [31,32].

### 4.3. Clinical Implications

From a clinical perspective, these findings suggest that prognostic reassessment in sepsis may benefit from moving beyond isolated admission biomarker values toward a biologically informed 48 h reassessment approach. In our cohort, PCT clearance emerged as the most informative routine biomarker component beyond SOFA, while NLR change appeared to provide complementary prognostic information in exploratory combined modeling. Further, 48 h LAC also provided incremental information beyond SOFA in the primary models, suggesting that persistent lactate elevation after early treatment remains clinically relevant; however, it did not retain independent significance when combined with SOFA and PCT clearance. Importantly, these markers are not experimental or resource-intensive, but are rapidly available and already embedded in routine care in many hospitals, which supports their suitability for further validation.

Although our results should not be interpreted as sufficient to support immediate changes in management algorithms, they do suggest that combining a clinical severity score with selected early reassessment biomarkers may offer a more nuanced representation of early sepsis trajectory than reliance on baseline measurements alone. If confirmed in larger and externally validated cohorts, these reassessment variables may inform future work on early prognostic reassessment during the first 48 h of hospitalization, when early biological response and organ dysfunction are still evolving and prognostic reassessment may be particularly informative.

### 4.4. Limitations and Future Directions

Several limitations should be acknowledged.

First, because the principal biomarker variables required M2 sampling, the main analysis applies to patients who were alive and had complete biomarker reassessment data at 48 ± 6 h. The findings should therefore not be interpreted as admission-time prediction for all patients presenting with sepsis.

This was a single-center observational study with a relatively modest sample size and a limited number of outcome events, which constrains statistical power and may reduce the stability and generalizability of the fitted models. In addition, 28 eligible patients declined informed consent before inclusion, which may have introduced participation bias and should be considered when interpreting the generalizability of the cohort.

Our findings should be interpreted cautiously. The analysis was exploratory, single-center, and based on a modest number of outcome events. Although model construction was clinically guided and intentionally parsimonious, it was partly informed by observed descriptive and univariate biomarker signals, which may introduce selection bias and optimism in the estimated model performance. In addition, several candidate biomarker models were evaluated, and no external validation was performed. Treatment-process variables, infection-source heterogeneity, and center-specific care pathways may also have influenced biomarker trajectories and outcomes. Therefore, the findings should be viewed as hypothesis-generating and require validation in larger independent cohorts.

The analysis was performed using a complete-case approach, and patients with incomplete data were excluded; although this improved internal consistency of the analytic dataset, it may also have introduced selection bias.

Source-control procedures and the timing or adequacy of source control were not systematically adjudicated in the predefined dataset. Therefore, we could not evaluate how source-control success influenced biomarker kinetics, particularly PCT clearance.

Although AKI and renal replacement therapy were recorded descriptively, the study was not designed to specifically evaluate whether renal dysfunction modified the association between PCT clearance and mortality. Because renal dysfunction may influence PCT kinetics, future studies should assess the prognostic value of PCT clearance stratified by AKI status and renal replacement therapy.

The exploratory combined biomarker models, particularly the model including SOFA, PCT clearance, and NLR change, should be interpreted with caution. Although the models were intentionally parsimonious, the number of outcome events was limited and several candidate biomarker models were evaluated; therefore, model performance estimates, including AUCs and regression coefficients, may be optimistic. These models were not externally validated and should be regarded as exploratory rather than definitive clinical tools.

Because the study was designed to evaluate dynamic biomarkers beyond admission SOFA, standardized 48 h SOFA reassessment was not collected a priori and therefore excluded from analysis. Consequently, the present analysis demonstrates incremental information beyond admission SOFA, but cannot determine whether these biomarkers add prognostic value beyond serial organ dysfunction reassessment or SOFA change over the same interval.

Another relevant consideration is the recent development of SOFA-2, an updated organ dysfunction score intended to better reflect contemporary critical-care practice, including current organ-support interventions and revised organ-system thresholds [33]. The present study was designed and conducted using the original SOFA framework, which remains the basis of the Sepsis-3 definition applied for patient inclusion. Therefore, we did not recalculate prognostic models using SOFA-2. Future validation studies should assess whether dynamic biomarkers such as PCT clearance and NLR change retain incremental prognostic value when combined with SOFA-2 or with serial SOFA-2 trajectories.

Finally, our focus on accessible routine biomarkers strengthens bedside relevance but does not exclude the possibility that emerging host-response markers could further improve prognostic performance in future studies.

Accordingly, future research should aim to validate these findings in larger, multicenter cohorts, assess their robustness across different sepsis populations and treatment settings, and determine whether models combining clinical severity with dynamic routine biomarkers retain their performance when tested prospectively in independent datasets.

Considering that our study was not designed to develop a deployable individual-level prediction model, we did not perform a full prediction-model performance assessment, including calibration plots, calibration intercepts, calibration slopes, or Brier scores. Future validation studies should include these metrics before clinical implementation is considered. Additional work should also explore whether clinically actionable thresholds for PCT clearance or NLR change can be defined without sacrificing the predictive value preserved by continuous modeling, and whether these markers can be integrated into simple serial reassessment strategies suitable for routine bedside use.

## 5. Conclusions

In this prospective single-center cohort, routine biomarker reassessment at approximately 48 h provided exploratory prognostic information beyond admission SOFA among patients with sepsis who were alive and had complete M1 and M2 biomarker data. PCT clearance showed the clearest incremental association with 28-day mortality, while NLR change provided complementary information in combined modeling. Persistent 48 h lactate elevation was also informative, whereas lactate clearance was not independently associated with mortality. These findings support further investigation of simple routine biomarker reassessment strategies, but they should be interpreted as hypothesis-generating. The models were not developed or validated as clinical risk calculators and require confirmation in larger cohorts using an explicit landmark framework, serial organ dysfunction measures, calibration assessment, and external validation.

## Figures and Tables

**Table 1:** Baseline and 48 h Clinical and Biomarker Characteristics According to 28-day Mortality.

Variable	Total Cohort (n = 126)	Survivors (n = 82)	Non-Survivors (n = 44)	*p*-Value
Age, years [IQR]	75 [64–80]	75 [63–79]	76 [67–84]	0.037
Male sex, n (%)	56 (44.4)	41 (50.0)	15 (34.1)	0.127
SOFA at admission [IQR]	5 [4–7]	5 [4–6]	7 [5–8]	<0.001
Glasgow Coma Scale at admission [IQR]	13 [11–15]	13 [11–15]	12 [11–14]	0.102
CCI [IQR]	6.0 [5.0–7.0]	6.0 [4.2–7.0]	7.0 [5.0–8.0]	0.075
WBC M1, ×10^3^/uL [IQR]	14.65 [11.12–20.54]	15.98 [11.58–21.68]	13.91 [9.99–18.85]	0.101
NLR M1 [IQR]	8.11 [6.52–9.49]	7.11 [6.15–8.92]	9.28 [8.11–10.60]	<0.001
Hb M1, g/dL [IQR]	10.83 [9.40–11.68]	10.90 [10.20–11.74]	10.50 [8.46–11.10]	0.103
Platelets M1, ×10^3^/uL [IQR]	193.0 [146.0–254.0]	197.3 [130.0–254.0]	187.9 [160.9–253.8]	0.872
Creatinine M1, mg/dL [IQR]	1.33 [0.76–2.13]	1.15 [0.71–2.12]	1.69 [0.81–2.27]	0.509
CRP M1, mg/L [IQR]	198.10 [96.40–273.92]	194.37 [115.76–257.36]	214.90 [69.92–293.20]	0.600
PCT M1, ng/mL [IQR]	2.46 [0.62–8.51]	3.00 [0.62–12.31]	1.13 [0.63–3.94]	0.198
LAC M1, mmol/L [IQR]	3.65 [2.65–4.97]	3.68 [2.55–4.97]	3.68 [3.13–5.01]	0.521
Glasgow Coma Scale M2 [IQR]	14 [12–15]	14 [12–15]	13 [12–14]	0.172
WBC M2, ×10^3^/uL [IQR]	13.30 [11.40–14.25]	13.30 [11.40–14.25]	12.35 [11.16–13.30]	0.172
NLR M2 [IQR]	7.00 [6.10–8.84]	6.28 [5.56–7.69]	8.83 [7.07–10.34]	<0.001
Hb M2, g/dL [IQR]	10.62 [9.00–11.66]	10.90 [9.30–11.66]	9.55 [8.30–11.47]	0.214
Platelets M2, ×10^3^/uL [IQR]	190.4 [131.5–265.2]	196.2 [142.0–267.0]	187.8 [117.0–242.8]	0.270
Creatinine M2, mg/dL [IQR]	1.12 [0.75–1.95]	0.93 [0.70–1.87]	1.42 [0.79–2.19]	0.059
CRP M2, mg/L [IQR]	94.77 [39.84–157.86]	80.97 [40.51–133.85]	139.33 [41.58–177.22]	0.065
PCT M2, ng/mL [IQR]	0.83 [0.31–5.66]	0.82 [0.28–5.09]	0.86 [0.41–5.92]	0.554
LAC M2, mmol/L [IQR]	3.48 [2.29–5.54]	3.18 [2.12–4.80]	4.19 [3.02–6.39]	0.013

Footnote: Continuous variables are presented as median [IQR], and categorical variables as n (%). *p*-values were calculated using the Mann–Whitney U test for continuous variables and the Pearson chi-square test for categorical variables. M1 = first biomarker measurement at admission or sepsis recognition; M2 = repeat biomarker measurement 48 ± 6 h after M1; CCI = Charlson Comorbidity Index; SOFA = Sequential Organ Failure Assessment; WBC = white blood cell count; NLR = neutrophil-to-lymphocyte ratio; Hb = hemoglobin; CRP = C-reactive protein; PCT = procalcitonin.

**Table 2:** Univariate Logistic Regression Analysis for 28-day Mortality.

Predictor	Scale/Comparison	OR	95% CI	*p*-Value
Age	per 1 year	1.03	0.97–1.09	0.066
Male sex	male vs. female	0.52	0.24–1.10	0.089
SOFA	per 1 point	1.56	1.25–1.93	<0.001
CCI	per 1 point	1.25	0.98–1.59	0.071
NLR M1	per 1 unit	1.86	1.45–2.39	<0.001
CRP M1	per 10 mg/L	1.01	0.98–1.05	0.564
PCT M1	per 1 ng/mL	0.99	0.97–1.00	0.133
LAC M1	per 1 mmol/L	1.10	0.96–1.26	0.183
NLR M2	per 1 unit	2.05	1.56–2.69	<0.001
CRP M2	per 10 mg/L	1.05	1.01–1.10	0.028
PCT M2	per 1 ng/mL	0.99	0.97–1.02	0.498
LAC M2	per 1 mmol/L	1.25	1.05–1.49	0.014
NLR change	per 10% increase	0.66	0.45–0.97	0.033
CRP clearance	per 10% increase	0.95	0.88–1.03	0.203
PCT clearance	per 10% increase	0.89	0.83–0.96	0.001
LAC clearance	per 10% increase	0.94	0.87–1.01	0.110

Footnote: All models were univariate logistic regression models with 28-day mortality as the dependent variable (1 = death, 0 = survival). ORs for continuous predictors are unstandardized. CRP was modeled per 10 mg/L increase. Clearance variables were modeled per 10% increase and calculated as [(M1 − M2)/M1] × 100; higher values therefore indicate a greater decline from baseline. Sex was modeled as male vs. female, with female as the reference category. PCT was modeled per 1 ng/mL increase, LAC per 1 mmol/L increase, and clearance variables per 10% increase.

**Table 3:** Primary Multivariable Prognostic Models for 28-day Mortality Beyond SOFA.

Model	Predictor	Coefficient (β)	OR (95% CI)	*p*-Value	AUC	95% CI for AUC	Nagelkerke R^2^	LR Test vs. Model 0
Model 0 (SOFA)	SOFA (per 1 point)	0.443	1.56 (1.25–1.93)	<0.001	0.740	0.636–0.832	0.206	Reference
Model 1 (SOFA + CRP M2)	SOFA (per 1 point)	0.416	1.52 (1.22–1.88)	<0.001	0.751	0.645–0.849	0.225	0.152
	CRP M2 (per 10 mg/L)	0.034	1.03 (0.99–1.08)	0.154				
Model 2 (SOFA + PCT clearance)	SOFA (per 1 point)	0.457	1.58 (1.26–1.98)	<0.001	0.810	0.721–0.885	0.332	<0.001
	PCT clearance (per 10% increase)	−0.129	0.88 (0.81–0.95)	0.001				
Model 3 (SOFA + LAC M2)	SOFA (per 1 point)	0.417	1.54 (1.24–1.92)	<0.001	0.770	0.687–0.854	0.245	0.039
	LAC M2 (per 1 mmol/L)	0.199	1.22 (1.01–1.48)	0.043				
Model 4 (SOFA + NLR change)	SOFA (per 1 point)	0.454	1.58 (1.26–1.96)	<0.001	0.765	0.664–0.855	0.254	0.021
	NLR change (per 10% increase)	−0.463	0.63 (0.41–0.95)	0.029				

Footnote: Outcome was 28-day mortality within the 48 h landmark analytical cohort. CRP M2 was entered per 10 mg/L increase. PCT clearance was calculated as ((M1 − M2)/M1) × 100 and modeled per 10% increase. LAC M2 was entered per 1 mmol/L increase. NLR change was calculated as ((NLR M1 − NLR M2)/NLR M1) × 100 and modeled per 10% increase; higher values indicate greater early NLR decline. AUC confidence intervals were estimated by nonparametric bootstrap. Likelihood-ratio tests compare each augmented model with Model 0. These models were exploratory and were not internally validated for clinical risk prediction.

**Table 4:** Exploratory combined biomarker models for 28-day mortality beyond SOFA.

Model	Predictor	Coefficient (beta)	OR (95% CI)	*p*-Value	AUC	95% CI for AUC	Nagelkerke R^2^	LR Test vs. Model 2
Exploratory Model A	SOFA (per 1 point)	0.474	1.61 (1.27–2.03)	<0.001	0.821	0.733–0.895	0.378	χ^2^ = 5.72; *p* = 0.017
	PCT clearance (per 10% increase)	−0.132	0.88 (0.81–0.95)	0.001				
	NLR change (per 10% increase)	−0.525	0.59 (0.37–0.94)	0.026				
Exploratory Model B	SOFA (per 1 point)	0.441	1.55 (1.24–1.95)	<0.001	0.826	0.751–0.902	0.353	χ^2^ = 2.548; *p* = 0.110
	PCT clearance (per 10% increase)	−0.129	0.88 (0.81–0.95)	0.002				
	LAC M2 (per 1 mmol/L)	0.169	1.18 (0.96–1.46)	0.115				

## Data Availability

The original contributions presented in this study are included in the article. Further inquiries can be directed to the corresponding author.

## Abbreviations

The following abbreviations are used in this manuscript:

SOFA	Sequential Organ Failure Assessment
CRP	C-Reactive Protein
LAC	Lactate
PCT	Procalcitonin
NLR	Neutrophil-to-Lymphocyte Ratio
M1	First Measurement
M2	Second Measurement
CCI	Charlson Comorbidity Index
GCS	Glasgow Come Scale
